# Data on simulated interpersonal touch, individual differences and the error-related negativity

**DOI:** 10.1016/j.dib.2016.04.016

**Published:** 2016-04-13

**Authors:** Mandy Tjew-A-Sin, Mattie Tops, Dirk J. Heslenfeld, Sander L. Koole

**Affiliations:** a Department of Clinical Psychology, Vrije Universiteit Amsterdam, Van der Boechorststraat 1, 1081 BT Amsterdam, The Netherlands; b Department of Cognitive Psychology, Vrije Universiteit Amsterdam, Van der Boechorststraat 1, 1081 BT Amsterdam, The Netherlands

**Keywords:** Touch, Error-related negativity, EEG, Individual differences

## Abstract

The dataset includes data from the electroencephalogram study reported in our paper: ‘*Effects of simulated interpersonal touch and trait intrinsic motivation on the error-related negativity’* (doi:10.1016/j.neulet.2016.01.044) (Tjew-A-Sin et al., 2016) [1]. The data was collected at the psychology laboratories at the Vrije Universiteit Amsterdam in 2012 among a Dutch-speaking student sample. The dataset consists of the measures described in the paper, as well as additional (exploratory) measures including the Five-Factor Personality Inventory, the Connectedness to Nature Scale, the Rosenberg Self-esteem Scale and a scale measuring life stress. The data can be used for replication purposes, meta-analyses, and exploratory analyses, as well as cross-cultural comparisons of touch and/or ERN effects. The authors also welcome collaborative research based on re-analyses of the data. The data described is available at a data repository called the DANS archive: http://persistent-identifier.nl/?identifier=urn:nbn:nl:ui:13-tzbk-gg.

**Specifications Table**

**Table:** 

Subject area	*Psychology*
More specific subject area	*Cognitive neuroscience*
Type of data	*SPSS .sav data (or.csv) and syntax file, .cnt, .ev*2 *event files, .wksp Brain Vision Analyzer Workspace and .ehst*2*/hfinf*2 *History files*
How data was acquired	*Questionnaire answers were acquired using Adobe Authorware. The EEG recording was acquired using Neuroscan acquisition software* (*Compumedics Neuroscan, Hamburg, Germany*) *and analyzed using Brain Vision Analyzer software* (*Brain Products, Gliching, Germany*).
Data format	*Filtered, analyzed, reverse scored and raw.*
Experimental factors	*The study had a within-subjects factorial design in which participants completed two sessions of a Go/No-Go task, one while holding a teddy bear and one while holding a same-sized cardboard box* (*order was counterbalanced*).
Experimental features	*The main outcome measures were performance and ERNs during the Go/No-Go task. We also measured individual differences such as trait intrinsic motivation, self-esteem, life stress etc.*
Data source location	*Vrije Universiteit Amsterdam, The Netherlands.*
Data accessibility	*Data is within this article and at the DANS data repository at the following permanent link:* http://persistent-identifier.nl/?identifier=urn:nbn:nl:ui:13-tzbk-gg.

**Value of the data**

● The data allows exploration of simulated interpersonal touch effects on ERN amplitudes and band frequencies during Go/No Go task performance.
● The data may be used to explore how individual differences in personality, motivation, self-esteem, life stress and connectedness to nature are related to EEG brain activity and Go/No Go task performance.
● The data can be used for re-analyses, replication purposes, and meta-analyses on ERN effects in relation to contextual factors and individual differences.

## 1 Data

The dataset described consists of a SPSS.sav data (or.csv) and syntax file with the prepared questionnaire and EEG data, .cnt files containing the raw EEG recordings per participant, .ev2 event files that contain participants׳ responses during the Go/No-Go task, and a .wksp Brain Vision Analyzer Workspace file and .ehst2/hfinf2 History files used to filter and organise the raw data.

## 2 Experimental design, materials and methods

The experimental design and methods are largely described in the published article [1], which also contains details about the Go/No-Go task, the EEG recordings and the processing of the raw data. Thus, we expand here only on the additional measures in the dataset that were not described in the paper. The dataset is available at the following link: http://persistent-identifier.nl/?identifier=urn:nbn:nl:ui:13-tzbk-gg*.*

### 2.1 Participants and design

None of the participants had a history of neurological or psychiatric disease. The study was conducted in accordance with the Code of Ethics of the World Medical Association. All participants gave written informed consent prior to the study. Our dataset consisted of 20 participants (16 women, 4 men; average age: 20). The study had a within-subjects factorial design in which participants completed two sessions of a Go/No-Go task, one while holding a teddy bear on their lap and one while holding a similarly sized cardboard box. The main outcome measures were performance and ERNs during the task.

### 2.2 Procedure and materials

The questionnaire portion of the experiment was conducted behind a computer in a soundproof chamber and the EEG recordings were made in a room-sized Faraday cage. We informed participants that we were investigating the effects of distracting objects on cognitive performance. Participants completed a set of questionnaires which included the Action Control Scales [2] containing the 12-item Persistence subscale that we used to measure trait intrinsic motivation. Participants answered the Action Control Scales by choosing between two answer options per item, reflecting either a high or a low intrinsic motivation response. The number of action-oriented responses on the Persistence subscale was our index of trait intrinsic motivation.

We also measured the following: pre-manipulation mood (shortened 32-item Profile of Mood States on a scale of 1–9) [3], self-esteem (10-item Rosenberg Self-esteem Scale on a scale of 1–9) [4], personality (100-item Five-Factor Personality Inventory on a scale of 1–5) [5], life stress (adapted 8-item version of the Demands and Threats subscales on a scale of 1–5) [6], [7], trait levels of feeling connected to nature (14-item Connectedness to Nature Scale on a scale of 1–5) [8], and quality of participants׳ living space (e.g., nature or urban environment, noise pollution items rated on a scale of 1–9).

After completing the questionnaires, we prepared the participant for EEG recording. Participants were informed that they would hold different objects on their lap during the task. We continuously measured EEG while participants completed a Go/No-Go task, which also included some practice trials. For the actual task, participants completed two sessions in random order, one while holding an 80 cm teddy bear and one while holding a cardboard box. Finally, participants were asked for some biographical information (e.g., sex, age), debriefed and thanked.
